# Changes in Holstein cow milk and serum proteins during intramammary infection with three different strains of *Staphylococcus aureus*

**DOI:** 10.1186/1746-6148-7-51

**Published:** 2011-09-01

**Authors:** Yunee Kim, Heba Atalla, Bonnie Mallard, Claude Robert, Niel Karrow

**Affiliations:** 1 Center for Genetic Improvement of Livestock, Department of Animal and Poultry Science, University of Guelph, Guelph, Ontario, N1G 2W1, Canada; 2 Department of Pathobiology, Ontario Veterinary College, University of Guelph, Guelph, Ontario, N1G 2W1, Canada; 3 Centre de recherche en biologie de la reproduction, Pavillon des Services, INAF, Université Laval, Québec, G1V 0A6 Canada

## Abstract

**Background:**

*Staphylococcus aureus *is one of the most prevalent pathogens to cause mastitis in dairy cattle. Intramammary infection of dairy cows with *S. aureus *is often subclinical, due to the pathogen's ability to evade the innate defense mechanisms, but this can lead to chronic infection. A sub-population of *S. aureus*, known as small colony variant (SCV), displays atypical phenotypic characteristics, causes persistent infections, and is more resistant to antibiotics than parent strains. Therefore, it was hypothesized that the host immune response will be different for SCV than its parental or typical strains of *S. aureus*. In this study, the local and systemic immune protein responses to intramammary infection with three strains of *S. aureus*, including a naturally occurring bovine SCV strain (SCV Heba3231), were characterized. Serum and casein-depleted milk cytokine levels (interleukin-8, interferon-γ, and transforming growth factor-β1), as well as serum haptoglobin concentrations were monitored over time after intramammary infection with each of the three *S. aureus *strains. Furthermore, comparative proteomics was used to evaluate milk proteome profiles during acute and chronic phases of *S. aureus *intramammary infection.

**Results:**

Serum IL-8, IFN-γ, and TGF-β1 responses differed in dairy cows challenged with different strains of *S. aureus*. Changes in overall serum haptoglobin concentrations were observed for each *S. aureus *challenge group, but there were no significant differences observed between groups. In casein-depleted milk, strain-specific differences in the host IFN-γ response were observed, but inducible IL-8 and TGF-β1 concentrations were not different between groups. Proteomic analysis of the milk following intramammary infection revealed unique host protein expression profiles that were dependent on the infecting strain as well as phase of infection. Notably, the protein, component-3 of the proteose peptone (CPP3), was differentially expressed between the *S. aureus *treatment groups, implicating it as a potential antimicrobial peptide involved in host defense against *S. aureus *intramammary infection.

**Conclusions:**

Intramammary infection of dairy cattle with *S. aureus *causes an up-regulation of serum and milk immune-related proteins, and these responses vary depending on the infecting strain.

## Background

Mastitis, an inflammatory condition of the mammary gland, typically arises as a result of intramammary infections. In lactating dairy cattle, mastitis results in considerable monetary losses to the dairy industry due to depressed milk yield and quality, reduced reproductive performance, treatment, and pre-mature culling [1,2]. *Staphylococcus aureus *is a Gram-positive, opportunistic, and contagious bacterium that is one of the most prevalent causes of mastitis in both humans and cattle [3]. Although infection can result in obvious clinical mastitis, *S. aureus *often evades the immune response resulting in a sub-clinical chronic infection that can persist for the life of the animal [4]. Classically, *S. aureus *is characterized as an extracellular pathogen [5]. However, an increasing number of studies elude to its ability to invade and survive within various host cell types, including mammary epithelial cells, neutrophils, and macrophages [6-8]; this may contribute to its persistence in various *S. aureus-*induced diseases [7]. Small colony variants (SCVs) of *S. aureus *may be particularly endowed for persistent survival by residing in host cells as they are characterized by slow-growth and atypical colony morphology, and are often over-looked by standard laboratory assays [9]. It has been previously demonstrated that SCVs can survive within professional and non-professional phagocytic cells [8,10], are more persistent than typical *S. aureus *strains [11], and are difficult to treat with conventional antibiotics [9].

Given that different *S. aureus *strains appear to utilize unique infection and survival strategies, we hypothesized that the host immune response will also vary in accordance with the infecting strain. To elucidate potential differences in host responses to *S. aureus *infection, the present study was carried out to investigate the kinetic response of key cytokines in serum and casein-depleted milk, as well as serum haptoglobin over a 21-day period following intramammary infection with three strains of *S. aureus*. Additionally, a comparative proteomics platform was used to resolve the proteome of casein-depleted milk during acute and chronic phases of infection, and to identify differentially expressed proteins with potential immunomodulating effects. The *S. aureus *strains used for intramammary infection were: the SCV Heba3231, a naturally occurring SCV isolated from a dairy cow with history of chronic mastitis with atypical phenotypic properties [10], the 3231 parent strain displaying typical morphological and biochemical properties [10], and Newbould 305 (ATCC 29740), a teat skin strain that causes clinical bovine mastitis [12,13] that is frequently used as a model for studying *S. aureus*-induced mastitis.

## Results

### Serum IL-8, IFN-γ, TGF-β1, and haptoglobin response to *S. aureus *intramammary infection

**Figure 1:**
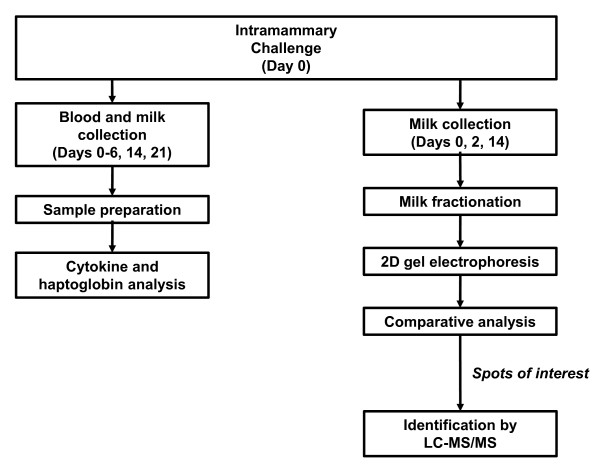
**Flow chart of study outline**.

**Figure 2:**
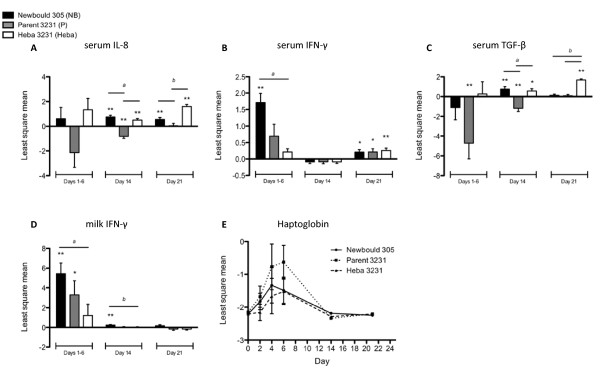
**Effects of intramammary infection with Newbould 305, Parent 3231, or Heba 3231 on cytokine and haptoglobin expression**. Least square mean of the log-transformed values ± standard error of the area under the curve for A) serum IL-8, B) serum IFN-γ, C) serum TGF-β, and D) milk IFN-γ on days 1-6 pi are shown. Least square mean of the log-transformed values ± standard error are shown for day 14 and day 21 pi. Significantly increased or decreased cytokine levels relative to baseline (day 0) values are denoted by asterisks (P < 0.05* and P < 0.01**). ^a,b ^indicate significant differences in cytokine levels between treatment groups (P < 0.05). E) Serum haptoglobin least square mean of log-transformed values ± standard error. Significant changes in serum haptoglobin concentration occurred over time for each challenge group (P < 0.01), but not at any individual time point, and no significant treatment differences were observed.

Lactating dairy cattle were challenged with one of three *S. aureus *strains and the local and systemic host immune responses were characterized by monitoring cytokine kinetics and using a proteomics approach to identify proteins that may play a role in host defense (Figure 1). Significant IL-8 expression in the serum was not evident for any of the challenge groups until day 14 post-infection (pi) (Figure 2A). Animals challenged with Newbould 305 and Heba 3231 exhibited significantly higher IL-8 expression on days 14 and 21 pi relative to levels on day 0 (P < 0.01). Interestingly, animals challenged with Parent 3231 showed a significant decrease in IL-8 expression on day 14 (P < 0.01), and no significant change on day 21 pi, relative to day 0. Significant differences in IL-8 expression between the three challenge groups were observed on days 14 and 21 pi, with both Newbould 305 and Heba 3231 challenge groups having significantly higher IL-8 expression than the Parent 3231 group (P < 0.01). Milk IL-8 levels were not statistically significantly different from baseline (day 0) levels at any time during the study (data not shown).

Serum concentrations of IFN-γ were significantly up-regulated in the Newbould 305 challenge group during days 1-6 pi relative to serum IFN-γ levels at day 0 (P < 0.01) (Figure 2B). On day 21 pi however, all challenge groups showed significant increases in IFN-γ expression compared to day 0 (Newbould 305 and Parent 3231, P < 0.05; Heba 3231, P < 0.01). Significant differences between challenge groups occurred on days 1-6 pi, whereby animals challenged with Newbould 305 had significantly higher serum IFN-γ levels compared to animals challenged with Heba 3231 (P < 0.01).

Serum TGF-β1 expression was significantly down-regulated in animals challenged with Parent 3231 throughout days 1-6 and 14 pi relative to day 0 (P < 0.01) (Figure 2C). In contrast, on day 14 pi, the Newbould 305 and Heba 3231 challenge groups had significantly up-regulated expression (P < 0.01 and P < 0.05, respectively). On day 21 pi, the Heba 3231 challenge group was the only group to have significantly elevated expression of TGF-β1 compared to levels at day 0 (P < 0.01). Significant differences in TGF-β1 expression between the three challenge groups were observed on day 14 pi, with Newbould 305 and Heba 3231 groups having significantly higher TGF-β1 levels compared to the Parent 3231 group (P < 0.01), and on day 21 pi, with the Heba 3231 group having significantly higher TGF-β1 expression compared to either Newbould 305 or Parent 3231 groups (P < 0.01). Milk TGF-β1 levels were not statistically significantly different from baseline at any time during the study period (data not shown).

Analysis of serum revealed significant elevations in haptoglobin concentrations within the first 24 hours pi for all three challenge groups (P < 0.01) (Figure 2E); however, linear and orthogonal polynomial contrasts did not reveal any significant differences in haptoglobin responses between any of the challenge groups.

### Milk IL-8, IFN-γ and TGF-β1 response to *S. aureus *intramammary infection

Significant changes in IL-8 and TGF-β1 expression were not observed in casein-depleted milk for any of the challenge groups at any time, when compared to levels on day 0 (data not shown). In contrast, IFN-γ concentrations were significantly increased in animals challenged with *S. aureus*, but the response varied with the infection strain (Figure 2D). For example, IFN-γ concentrations were increased in response to Newbould 305 on days 1-6 and day 14 pi relative to levels on day 0 (P < 0.01), but not day 21 pi. The Parent 3231 challenge group also showed a significant increase in IFN-γ expression on days 1-6 pi (P < 0.05). No significant change in IFN-γ expression was seen for the Heba 3231 challenge group at any sampling time. Significant differences between groups occurred on days 1-6 and day 14 pi, where the Newbould 305 challenge group had higher IFN-γ expression compared to the Heba 3231 group (P < 0.05).

### Milk proteome profiling following intramammary infection with *S. aureus*

Milk proteome analyses by 2-dimensional gel electrophoresis (2DE) revealed 29 protein spots that were differentially expressed on days 2 or 14 pi, relative to expression on day 0 (P < 0.05) (Table 1), with up-regulated expression occurring most frequently. In the Newbould 305 challenge group, there were 12 and 1 protein spots that were significantly induced on days 2 and 14 pi, respectively. In the group challenged with the Parent 3231 strain, 12 and 8 protein spots were significantly induced on days 2 and 14 pi, respectively. Lastly, for the Heba 3231 challenge group, no proteins were induced on day 2 pi, however, 8 protein spots were significantly induced on day 14.

**Table 1:** Change in milk protein expression on 2 and 14 days post-intramammary infection with different *S.aureus *strains

**Spot I.D**.	Trmt*Day	LSM ± SEM	p-vlaue	Direction
11	P*14	0.03 ± 0.01	0.0205	↑
	Heba*14	0.02 ± 0.01	0.0382	↑

12	P*2	0.05 ± 0.02	0.0127	↑

13	P*2	0.02 ± 0.01	0.0206	↑
	Heba*14	0.02 ± 0.01	0.024	↑

21	P*14	0.03 ± 0.01	0.0397	↑

33	P*2	0.05 ± 0.02	0.0056	↑

35	NB*2	0.02 ± 0.004	0.0041	↑
	Heba*14	0.01 ± 0.005	0.0467	↑

39 (CPP3)	P*2	0.02 ± 0.007	0.0476	↑
	P*14	0.01 ± 0.004	0.0032	↑
	Heba*14	0.01 ± 0.004	0.0154	↑

45	NB*2	0.01 ± 0.004	0.0122	↑

131	NB*2	0.01 ± 0.005	0.031	↑

141	NB*2	0.04 ± 0.01	0.0296	↑

212	P*2	0.01 ± 0.004	0.0038	↑
	Heba*14	0.01 ± 0.005	0.0408	↑

220	P*2	0.04 ± 0.01	0.0214	↑

	Heba*14	0.01 ± 0.005	0.0304	↑

222	NB*2	0.02 ± 0.009	0.0384	↑

227	P*2	0.008 ± 0.003	0.0127	↑

235 (CPP3)	P*2	0.03 ± 0.01	0.0241	↑
	P*14	0.01 ± 0.006	0.0492	↑
	Heba*14	0.02 ± 0.006	0.0127	↑

244	P*14	-0.008 ± 0.002	0.0016	↓

250	NB*2	0.007 ± 0.003	0.0417	↑
	NB*14	0.01 ± 0.005	0.0535	↑

251	P*2	0.04 ± 0.02	0.0449	↑
(β-casein precursor)	P*14	0.008 ± 0.002	0.0025	↑

341	P*14	0.02 ± 0.004	0.0006	↑

343	P*14	0.02 ± 0.009	0.0188	↑

346	NB*2	0.02 ± 0.01	0.0331	↑

349 (CPP3)	NB*2	0.01 ± 0.004	0.0084	↑

350	NB*2	0.02 ± 0.01	0.0423	↑

351	P*2	0.04 ± 0.01	0.0079	↑

	P*14	0.02 ± 0.01	0.0467	↑

352	P*2	0.01 ± 0.006	0.0411	↑

354	NB*2	-0.02 ± 0.004	< 0.0001	↓
(β-Lactoglobulin)	NB*14	-0.02 ± 0.007	0.0105	↓
	Heba*14	0.01 ± 0.007	0.0459	↑

355	NB*2	0.01 ± 0.005	0.0481	↑

358	NB*2	0.05 ± 0.02	0.0216	↑

359	NB*2	0.01 ± 0.005	0.0494	↑
	P*2	0.01 ± 0.005	0.0474	↑

NB, Newbould 305; P, Parent 3231; Heba, SCV Heba 3231

Trmt*Day denotes the treatment and day of the observed change in protein spot

CPP3, component 3 of the proteose peptone

Data are presented as the least square mean ± standard error of the mean (LSM ± SEM).

Direction indicates the up-or down-regulation of the protein spot on day 2 or 14 pi, compared to baseline.

Spot I.D. number refers to protein spot number assigned by the Melanie software. Spots that were excised and identified by LC-MS/MS are indicated in parentheses.

**Figure 3:**
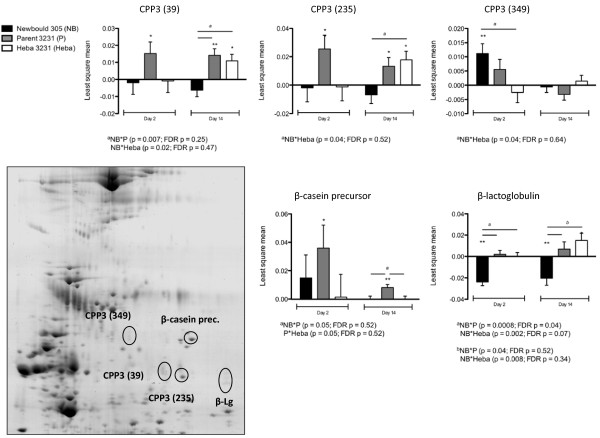
**Two-dimensional gel electrophoresis of milk proteins**. Proteins were resolved on a 13 cm Immobiline DryStrip, pH 3-10, followed by 12.5% SDS-PAGE. Circled spots are significantly different in protein expression between treatment groups as well as on day 2 or 14 pi, relative to day 0 values. Corresponding graphs detail the least square mean of the log transformed spot intensities ± SEM for each spot. Significant changes in protein expression levels relative to baseline (day 0) are denoted by asterisks (P < 0.05* and P < 0.01**). ^a,b ^significant differences in protein expression between treatment groups on day 2 or 14 pi with raw p-values and FDR-adjusted p-values presented (P < 0.05).

Of the 29 spots that were differentially expressed during the acute or chronic phases of infection, only 1 spot was significantly differentially expressed over time and between the *S. aureus *challenge groups based on the FDR-adjusted P-value (P < 0.05). This spot, and four other spots that were differentially expressed over time and between the treatment groups based on their raw P-values (P < 0.05), were excised and subjected to protein sequencing by liquid chromatography and tandem mass spectrometry (LC-MS/MS). Identification of these protein spots and their expression patterns is summarized in Table 2 and Figure 3. Three out of the 5 spots were identified as component-3 of the proteose peptone (CPP3). Two of the spots corresponding to CPP3 (spot # 39 and 235) were up-regulated in animals challenged with Parent 3231 on day 2 and 14 pi (P < 0.05), as well as in the Heba 3231 challenge group on day 14 pi, with treatment differences occurring on day 14 pi between Newbould 305 and Parent 3231 or Heba 3231 challenge groups (P < 0.05). In contrast, CPP3 (spot # 349) was only up-regulated in the Newbould 305 challenge group on day 2 pi (P < 0.01), with significant treatment differences occurring between the Newbould 305 and Heba 3231 challenge groups (P < 0.05).

**Table 2:** Identification of proteins differentially expressed in different *S.aureus *challenge groups 2 or 14 days post-infection

**Spot I.D**.	Protein	**NCBI Accession no**.	MS result^a^	Th. Mass (kDa)/pI^b^	Obs. Mass (kDa)/pI^b^
39	Component PP3	2007376A	98; 13	15.3/5.98	16/6.5

235	Component PP3	2007376A	115; 13	15.3/5.98	15/7

251	β-casein precursor	P02666	72; 8	25.1/5.26	20/7

349	Component PP3	2007376A	208; 39	15.3/5.98	19/6

354	β-lactoglobulin	CAA32835	109; 11	20.3/4.85	15/8

^a^Ion score is = 10*Log(P) where P is the probability that the observed match is a random event (ion scores > 47 are significant at p < 0.05);% coverage.

^b^Theoretical (Th.) and observed (Obs.) masses and isoelectric points (pI).

## Discussion

The current study aimed to monitor the host immune response to *S. aureus *intramammary infection using three unique strains, and to identify potential candidates for targeted therapies that may modulate the host defense to *S. aureus *challenge. Intramammary infection with different strains of *S. aureus *resulted in variable cytokine responses within the bovine mammary gland and circulation, supporting our hypothesis that different strains of *S. aureus *prompt the activation of host immune responses that are unique to the infecting strain.

Typically, an increase in milk somatic cell count (SCC) is indicative of an elevated and sustained inflammatory response caused by intramammary infection. During acute mastitis for example, neutrophil numbers in the mammary gland increase dramatically, constituting > 90% of milk somatic cells [14], whereas somatic cells in healthy glands contain < 22% neutrophils [15]. CXCL-8/IL-8 is the main tissue-derived chemoattractant for neutrophils [16,17]; however, our observations demonstrated that the bovine IL-8 response did not always parallel the somatic cell response during the different *S. aureus *intramammary infections. Somatic cell scores were significantly elevated throughout the study period, and peaked during the acute phase (days 2-5 pi) in all challenge groups (reported by Atalla *et al*., 2009 [18]), however, we did not observe significant induction of IL-8 in the casein-depleted milk at any point during the study period, and it was only detected in the serum on days 14 and 21 pi in animals challenged with Newbould 305 and Heba 3231. The lack of an IL-8 response in the milk is consistent with two previously reported independent studies carried out by Bannerman *et al*. (2004) and Riollet *et al*. (2000b) [19,20], suggesting that bovine IL-8 is not the only chemokine involved in trafficking neutrophils to the infected mammary gland. It is possible that other chemoattractants, such as complement cleavage factor 5a, IL-1β, TNF-α, CXCL1 and 3, and granulocyte-macrophage colony stimulating factor (GM-CSF) produced by epithelial cells, macrophages and T cells, also contribute to neutrophil trafficking to the mammary gland during intramammary infection [21-23]. For instance, neutrophil infiltration into the bovine mammary gland may be mediated by IFN-γ through the mechanism described by McLoughlin *et al*. (2008) [8] that involves CXC chemokines other than IL-8. Such chemokines may include CXCL1 and CXCL3, which are constitutively expressed in bovine milk [23]. In support of this, it has been demonstrated that IL-8 and GM-CSF are not active chemokines during lactation, whereas other factors such as IL-1β and TNF-α are active during any stage of lactation [22,24].

In the present study, IFN-γ was found to be highly expressed both locally, as indicated by levels in the milk, and systemically during days 1-6 pi in animals challenged with Newbould 305, which parallels previously reported results by Bannerman *et al*. (2004) [19]. Serum IFN-γ levels were significantly up-regulated in all challenge groups at day 21 pi compared to day 0. This finding may be associated with the promotion of an enhanced cell-mediated immune response that includes neutrophil and macrophage activation.

During the chronic phase of infection, differences in serum TGF-β1 expression between the three challenge groups were observed with notable increases being observed for the Parent 3231 and Heba 3231 groups. Milk TGF-β1 did not significantly differ in response between any of the challenge groups; however, a trend towards altered TGF-β1 expression in animals challenged with Newbould 305 was observed, whereby the cytokine levels reached a maximum at 48 hours pi (approximately 4000 pg/ml, data not shown). This observation is in line with a previous report of milk TGF-β1 kinetics upon Newbould 305 challenge in dairy cattle [25].

The role of TGF-β in the host response to intramammary infection is uncertain because TGF-β is a pleiotropic immunoregulatory cytokine, with both pro- and anti-inflammatory functions, depending on the location and activation state of the cells that it is interacting with [25]. This is exemplified by the cytokine's dual actions on macrophages. For instance, TGF-β suppresses tissue macrophage function and inflammation by down-regulating the production of chemokines and cytokines such as IFN-γ [26], up-regulating the expression of IL-1 receptor antagonist [27], and suppressing the production of cytotoxic reactive oxygen and nitrogen intermediates [28,29]. In contrast, TGF-β has also been shown to promote inflammation by inducing chemotaxis in human peripheral blood monocytes [30,31], enhancing their ability for transendothelial migration to sites of infection [32], and up-regulating the transcription of pro-inflammatory cytokines such as IL-1β and TNF-α. Furthermore, TGF-β has been shown to be an important factor for the differentiation of a subset of effector Th cells known as Th17 cells, which elicit inflammatory responses during certain pathogenic infections that are not sufficiently dealt with by Th1 or Th2 immunity [33]. Lastly, regulatory cells including Tregs and M2 macrophages also produce TGF-β in humans. These cells help to prevent the deleterious effects of prolonged or excessive host immune responses [34,35], and this process includes suppressing various components of the immune response such as antigen presentation by antigen presenting cells [36], and promoting tissue repair by increasing the deposition of extracellular matrix at the site of tissue injury, and promoting angiogenesis [37]. Since TGF-β was not observed locally in the present study, it is unlikely that it was involved in repair of the mammary gland epithelium.

There is evidence to suggest that *S. aureus *may be able to modulate the host immune response in part by promoting TGF-β expression; this could be a mechanism utilized by the Heba 3231 strain for example, to promote its survival within the host. In support of this, Staphylococcal superantigens (SAgs) have been demonstrated to induce the production of TGF-β by initiating the differentiation and expansion of Tregs that down-regulate the inflammatory response [38-40]. Indeed, TGF-β has been implicated in playing a role in immunosuppression by up-regulating the expansion and/or activation of anergic regulatory T cells in the presence of *S. aureus *SAgs, which may disrupt class II MHC antigen presentation by APCs [41], limiting cell-mediated immunity. Interestingly, this immunomodulatory mechanism is also employed by *Leishmania *as a means to suppress cell-mediated immunity and promote its intracellular survival [42,43]. Lastly, it should be noted that TGF-β expression may not necessarily denote its level of bioactivity, since a study by Kehrl *et al*. (1986) [44] demonstrated that human B cells largely express inactive TGF-β in response *S. aureus *Cowan strain I.

Overall, haptoglobin concentrations increased with time in all challenge groups during the first 6 days of the study period, and declined to initial levels by days 14 and 21 pi. However, no significant differences in serum haptoglobin levels were observed between the challenge groups. This may have been due to the chosen sampling times, as no samples were taken between days 6 and 14 pi. It has been previously demonstrated that haptoglobin is up-regulated during the acute phase response, and that it may be used to discriminate between acute and chronic inflammation in cattle [45,46]. The findings in the present study indicate that overall serum haptoglobin levels were not significantly different between any of the challenge groups, suggesting that haptoglobin is a sensitive marker of inflammation due to *S. aureus *but it lacks specificity to discriminate the host response to the three different strains of *S. aureus*.

It was previously demonstrated that two common mastitis-causing pathogens, *Escherichia coli *and *S. aureus*, elicit different immune responses and pathogenesis [19]. Here, we demonstrate that the variability in immune protein response is also strain-specific, at least in the context of *S. aureus*. Further studies investigating the profiles of other cytokines will add to the results of the current study. Studying IL-17 and IL-6 for instance would provide insight into the involvement of Th17 cells in *S. aureus *infections, as IL-17 is a Th17 cytokine and IL-6, along with TGF-β supports the differentiation of this Th subtype [47-49]. Furthermore, IL-6 is one of the major signals for the induction of the acute phase response [50], and may therefore compliment the present haptoglobin data. Monitoring chemoattractive factors, especially those under the regulation of TGF-β (i.e. IL-1β and TNF-α), would help delineate the pro- or anti-inflammatory role of TGF-β in intramammary infection with different strains of *S. aureus*. Although it would have been interesting to measure IL-1-β and TNF-α as well as other cytokines such as IL-4, IL-6, IL-12, and IL-10 that regulate the differentiation of these T-cell subpopulations, we had limited resources to carry out the cytokine analysis, and felt that inclusion of these cyctokies was beyond the scope of the current study as the aim was to note differences in host responses to different *S. aureus *strains, not to determine whether TGF-β is a pro- or anti-inflammatory cytokine.

In addition to seeing unique cytokine profiles in response to the different strains of *S. aureus*, the present study also found a number of milk proteins to be differentially expressed at various time-points in response to infection, including host defense proteins. *Staphylococcus aureus *insult to the mammary gland causes an inflammatory response that is characterized by an increase in vascular permeability, allowing exudates containing cells and proteins from the vasculature to enter the mammary gland [50]. Additionally, resident and recruited cells produce a number of proteins that target the pathogen and contribute to the restoration of homeostasis [50]. Therefore, the proteome of the milk may drastically change in response to pathogenic stimuli, and analysis of these changes may offer insight into the host response during mastitis. Indeed, the present study demonstrated changes in the milk proteome during intramammary infection with *S. aureus*. In order to assess protein expression in milk during the acute and chronic phases of infection, samples were evaluated on days 2 and 14 pi, respectively. Most proteins were up-regulated following Newbould 305 and Parent 3231 challenges, especially on day 2 pi during the acute phase of infection. On day 14 pi, the number of up-regulated proteins was dramatically reduced in the Newbould 305 and Parent 3231 challenge groups, as compared to day 2 pi. Results from this study indicate that differences in milk protein expression also occur as a result of intramammary infection with different strains of *S. aureus*. On day 2 pi for example, challenges with Newbould 305 and Parent 3231 induced 12 milk proteins each, relative to day 0. In contrast, significant changes in milk protein expression were not observed in response to challenge with Heba 3231 at this time. On day 14 pi however, 8 milk proteins were induced by challenge with the Parent 3231 strain and only 1 protein was induced by Newbould 305, whereas 8 proteins were significantly induced by the Heba 3231 challenge. These results are indicative of the acute pathogenic nature of the Parent 3231 and Newbould 305 strains and the unique phenotypic properties of SCVs, which may elicit a milder host response [18]. They suggest a highly active initial host response to challenges with the Parent 3231 and Newbould 305 strains, then perhaps resolution or establishment of chronic infection by day 14 pi. This corresponds with the somatic cell score data from these same cows presented by Atalla *et al*. (2009) [18], whereby the number of somatic cells in the milk during the chronic phase of infection still remained significantly up-regulated relative to day 0. Interestingly, we were not able to detect an initial host response to the SCV strain, but the induction of protein expression was demonstrated on day 14 pi. This delayed and relatively low level of host response corresponds to the ability of the Heba 3231 strain to avoid recognition and persist within host cells [10]. Future studies should include later time points in order to determine if Heba 3231 continues to up-regulate milk protein expression.

Two of the five spots corresponded to the most abundant milk proteins - β-casein precursor and β-lactoglobulin. This finding is likely a result of incomplete depletion of these highly abundant proteins. Nonetheless, the depletion protocol was found to be sufficient to yield a comprehensive view of the milk proteome. Of the five protein spots that were subjected to identification by LC-MS/MS, only one protein, CPP3 also known as lactophorin, was implicated in host defense. Component 3 of the proteose peptone is a phosphoglycoprotein uniquely expressed in the mammary gland of lactating dairy cattle and can be found in bovine milk whey. It has been previously suggested to have several functions that include antimicrobial activity, inhibition of lipolysis, mitogenic activity, and immunostimulation [51-54]. It consists of seven polypeptide components, ranging in molecular weight from 17 to 67 kDa [55,56]. In the 2DE gels, it occurred at three different locations ranging in mass from 15-21 kDa and isoelectric point (pI) from 6-7 (Figure 3), which may correspond to its various components. In one study, the antimicrobial activity of CPP3 was investigated using a synthetic peptide called lactophoricin consisting of the 113-135 region of C-terminal of CPP3 [51]. Lactophoricin interacts with membrane phospholipids and forms voltage-dependent channels. Thus, it has been hypothesized that it may also be involved in the pore forming of natural lipid bilayers such as bacterial membranes [51,57]. Indeed, Campagna's results demonstrated that this peptide has inhibitory-growth effects on a number of Gram-positive and Gram-negative bacteria, with a more pronounced inhibitory effect on Gram-positive bacteria, including *S. aureus *[51].

## Conclusions

The present study demonstrates the complexity of the host immune protein response to various strains of *S. aureus*. Intramammary infection with different strains of *S. aureus*, elicited unique host immune protein responses, as indicated by differential expression of the cytokines IL-8, TGF-β, and IFN-γ in circulation and IFN-γ in casein-depleted milk. In general, cytokines displayed heightened responses in the serum compared to the casein-depleted milk. Interestingly, the SCV strain, Heba 3231, showed increased levels of serum IL-8, IFN-γ, and TGF-β occurring during the later stages of infection (day 21 pi). Whether this pattern of induction continues in later time points has yet to be determined. Using a proteomic approach, we have also demonstrated that intramammary infection with three different strains of *S. aureus *elicits differential protein expression in bovine milk during the acute and chronic phases of infection. Intramammary infection with Newbould 305 and Parent 3231 strains resulted in pronounced induction of protein expression on day 2 pi, while the Heba 3231 strain elicited no protein induction at this time. On day 14 pi, host protein activity was less pronounced for animals challenged with Newbould 305 and Parent 3231 strains, while some induction occurred in animals challenged with Heba 3231. Lastly, CPP3, a potential antimicrobial peptide that is uniquely expressed in the bovine mammary gland during lactation, appeared on our 2DE gels as three different spots, which were differentially expressed between the various *S. aureus *treatment groups. These results demonstrate that different strains of *S. aureus *affect the proteome of the milk in different ways. Of specific interest was the unique response to the SVC, Heba 3231 on day 21 pi. Therefore, investigating these changes in protein expression at later stages of infection is warranted. Overall, this study begins to shed light on the milk proteome and the changes that are elicited by intramammary infection with various strains of *S. aureus *and highlights an antimicrobial peptide, CPP3, which may have novel therapeutic potential.

## Methods

### Selection of animals

A detailed description of the selection criteria for cows used for intramammary infection has been previously described [18]. Briefly, healthy Holstein dairy cows (5 cows/treatment group) from the University of Guelph dairy herd and housed in the Ponsonby Research Station (Elora, Ontario, Canada) were selected for the studies under the following criteria: 1) quarter milk samples were negative for *S. aureus; *2) quarter milk samples contained ≤ 500 colony forming units (CFU) per ml of potential udder pathogens, other than *S. aureus*, that were mostly found in mixed culture; and 3) quarter milk samples had SCCs < 2 × 10^5 ^cells/ml. All cows were in their mid-to late-lactation and in the second or third trimester of gestation. They had no previous history of clinical mastitis and were in their first to fifth lactations. Animals were house in a tie-stall barn and fed a mixed ration formulated for the University dairy herd and milked twice daily. The study was approved by the University of Guelph Animal Care Committee.

### *S. aureus *intramammary challenge

The *S. aureus *intramammary challenges were carried out between January and May of 2007. A detailed description of the preparation of *S. aureus *cultures and intramammary challenge is described by Atalla *et al*. (2009) [18]. Cows were randomly assigned to one of three treatments and then challenged with either SCV Heba3231, 3231 parent strain, or the Newbould 305. Three quarters (right front, right hind, and left hind) were infused with ~1 × 10^3 ^CFU/ml of bacteria suspended in 5 ml of ice-cold pyrogen-free PBS through the teat canal using a syringe fitted with a 1.5 inch teat infusion canula. The left front quarter was infused with 5 ml of ice-cold pyrogen-free PBS, pH 7.4. The teats were then massaged in a dorsal direction and dipped in 1% iodine teat dip.

#### Sample collection

For this study, milk samples were only collected from the right hind quarter for analysis - the other two infected quarters and the saline quarter were used for sample collection by another group [18]. For collection, the teat was dipped in 1% iodine teat dip just prior to regular morning milking, fore-stripped by hand milking and the first two streams of milk were discarded. The teat was then wiped with a clean towel and scrubbed with gauze soaked in 70% isopropyl alcohol, and milk samples were subsequently collected into sterile 50 ml centrifuge tubes on day 0 before intramammary challenge, and days 1-6, 14, and 21 pi. Samples were kept at 4°C or on ice during transportation to the laboratory where they were immediately processed at 4°C. For the preparation of casein-depleted milk, whole milk samples were centrifuged at 1500 *g *and 4°C for 30 min. The top lipid layer was discarded and the remaining skim milk was stored at -20°C until ultracentrifugation. For ultracentrifugation, milk samples were thawed at 4°C for 15-17 hr, and centrifuged at 45,500 *g *at 4°C for 30 min. The resulting casein-depleted milk was recovered and stored at -20°C until used.

Approximately 10 ml of blood from the tail vein of each cow was collected into vacutainers (SST Gel and Clot Activator from BD Biosciences, Mississauga, Ontario, Canada) on the mornings of days 0-6, 14, 21, 28, and 35 pi. The samples were kept at 20°C for a maximum of 1 hour during transportation from the animal housing facility to the laboratory. Samples were then centrifuged at 1200 *g *at 20°C for 30 min to obtain sera and stored at -80°C until used.

### Analysis of IL-8, TGF-β1, and IFN-γ in serum and casein-depleted milk

Serum and milk cytokines were measured on days 0, 1, 2, 4, 6, 14, and 21 pi by ELISA. For IL-8, a commercially available human IL-8 ELISA kit (R&D Systems Inc., Minneapolis, Minnesota, USA) was used to measure bovine IL-8 in milk and serum samples according to the manufacturer's instructions with a slight modification. The antibodies used in the kit have been previously validated to cross-react with bovine IL-8 [58]. As the manufacturer's instructions were not optimized for use in milk, the sample incubation time for the analysis of IL-8 in casein-depleted milk was increased to 20 hr at 4°C. The inter- and intra-assay coefficients of variation for serum were 6% and ≤ 3%, respectively, and for the milk they were 11% and ≤ 3%, respectively. The limit of detection for this assay was 0.4 pg/ml.

Serum and milk IFN-γ concentrations were measured using a commercially available bovine IFN-γ ELISA kit (Mabtech Inc., Cincinnati, Ohio, USA) according to the manufacturer's instructions. As the manufacturer's instructions were not optimized for use in milk, the sample incubation time for the analysis of IFN-γ in casein-depleted milk was increased to 20 hr at 4°C. The inter- and intra-assay coefficients of variation for serum were 8% and < 4%, respectively, and for the casein-depleted milk they were 6% and ≤6%, respectively. The limit of detection for this assay was 9 pg/ml.

Lastly, a commercially available human TGF-β1 ELISA kit (R&D Systems Inc.) that has been previously validated to cross-react with bovine TGF-β1 by Ginjala and Pakkanen (1998) [59]. For activation of latent TGF-β in casein-depleted milk, 0.1 ml of undiluted casein-depleted milk was incubated with 0.1 ml of 2.5 N acetic acid/10 M urea for 10 min. Then 0.1 ml of 2.7 N NaOH/1 M HEPES was added to neutralize the reaction and the final solution was diluted 4-fold with Reagent Diluent Concentrate (R&D Systems, Inc.). Casein-depleted milk samples were assayed according to the manufacturer's instructions with the exception of the sample incubation time, which was increased to 20 hr at 4°C. For serum TGF-β1, serum samples were activated and diluted 10-fold with Reagent Diluent Concentrate (R&D Systems Inc.). Samples were then assayed according to the manufacturer's instructions. The inter- and intra-assay coefficients of variation for serum were 7% and ≤ 3%, respectively; for milk they were 6% and ≤ 3%, respectively. The limit of detection for this assay was 8 pg/ml.

### Haptoglobin

Serum haptoglobin was quantified in samples collected on days 0, 1, 2, 4, 6, 14, and 21 pi, at the University of Guelph Animal Health Lab (Guelph, Ontario). Spectrophotometric quantification was based on the method developed by Makimura and Suzuki (1982) [60], which utilizes differences in peroxidase activity in free hemoglobin versus hemoglobin/haptoglobin conjugate.

### Milk proteome profiling by 2 D gel electrophoresis

For the preparation of milk for 2DE, casein-depleted milk samples were subjected to β-lactoglobulin (β-Lactoglobulin) depletion by cyanogen bromide-activated Sepharose 4B conjugation with β-Lactoglobulin. In order to do this, a matrix was first prepared by washing CNBr-activated Sepharose powder (GE Healthcare, Québec, Canada) with 200 ml of 1 mM HCl for every 1 g of powder. The matrix was equilibrated with coupling buffer (0.1 M NaHCO_3 _containing 0.5 M NaCl, pH 8.3) by rotating on a sample mixer for 20 min at 20°C. The β-Lactoglobulin (Sigma-Aldrich, Oakville, Ontario, Canada) was dissolved in coupling buffer to a final concentration of 12 mg/ml β-Lactoglobulin-coupled gel matrix. The dissolved β-Lactoglobulin solution and the equilibrated gel medium were mixed for 2 hr at 20°C. The reaction was quenched with 1 M ethanolamine (pH 8.0) for 2 hr at 20°C. The mixture was centrifuged, and the supernatant was discarded without perturbing the β-Lactoglobulin-coupled gel medium. The coupled medium was washed with 0.1 M NaCl/HCl (pH 2) and then equilibrated with water for 20 min at 20°C, centrifuged for 1 min at 16,000 *g *to pellet the beads, and the supernatant discarded without perturbing the medium. The matrix was stored at 4°C in 20% ethanol until used.

For β-Lactoglobulin depletion, 1 × sample volume of sodium acetate buffer (pH 4.6) was added to the samples, mixed, and centrifuged at 16,000 *g *for 30 min at 4°C. The resulting supernatant was transferred to the CNBr-activated Sepharose 4B beads conjugated with β-Lactoglobulin, mixed on a rotating sample mixer for 20 min and centrifuged at 16,000 *g *to separate the β-Lactoglobulin-conjugated beads. The resulting supernatants were subjected to methanol-chloroform treatment to precipitate proteins. First, 2 × the sample volume of methanol and 1 × the sample volume of chloroform was added to the samples and proteins were allowed to precipitate for 1 hr at -20°C. The samples were centrifuged, the upper phase was discarded and another round of methanol and chloroform was added to the remaining samples and mixed. The samples were centrifuged and the supernatants were discarded, and the resulting protein pellets were solubilized to a concentration of 200 μg protein/250 μl in rehydration solution containing 8 M urea, 2% w/v CHAPS, 0.28% w/v DTT, 0.5% w/v IPG buffer (GE Healthcare), and bromophenol blue. Protein concentration was determined using a Nanodrop Spectrophotometer (Nanodrop Technologies, Wilmington, Delaware, USA).

For 2DE proteome profiling, the casein-depleted and β-Lactoglobulin-depleted milk samples were separated in the first dimension by isoelectric focusing (IEF) on 13 cm Immobiline DryStrips (pH 3-10; GE Healthcare) using the following protocol carried out at 20°C on an Ettan IPGphor IEF system (GE Healthcare). Strip rehydration was carried out for 2 hr then IEF was carried out at 30 V for 10 hr, 100 V for 1 hr, 300 V for 1 hr, 600 V for 1 hr, 1000 V for 1 hr, 3000 V for 1 hr, 4000 V for 2 hr, and 8000 V for 2 hr at 50 μA/strip. The strips were stored at -80°C until the second-dimension separation. For separation in the second dimension, strips were equilibrated for 15 min in equilibration buffer containing 1.5 M Tris-HCl (pH 8.8), 6 M Urea, 30% v/v glycerol, 2% w/v SDS, bromophenol blue, and 1% w/v DTT. The strips were cast in 0.5% w/v agarose on 12% SDS-PAGE gels and run at 30 mA/gel. Protein migration was stopped when the dye front reached the bottom of the gels, and fixation was carried out overnight in 50% methanol and 10% acetic acid. The gels were stained with RAPIDstain (G-Biosciences, Maryland Heights, Missouri, USA) following the manufacturer's instructions and scanned using ImageScanner (GE Healthcare).

Gels were analyzed using Melanie 7.0 software (GeneBio, Geneva, Switzerland). Protein spot intensities were automatically background-subtracted on a spot basis, whereby the lowest 10^th ^percentile pixel value on the spot boundary was excluded from all other pixel values within the spot boundary. Spots were matched based on a hierarchical matching structure, as suggested in the Melanie 7.0 user manual.

The five protein spots of interest on the 2DE gels were excised using a scalpel. Spot reduction, akylation, tryptic digestion, and mass spectrometric analysis were performed at McGill University and Genome Québec Innovation Centre (Montréal, Québec, Canada). Excised spots were subjected to reduction, cysteine-alkylation and in-gel tryptic digestion using an automated MassPrep Workstation (Micromass UK Ltd., Wythenshawe, Manchester, UK) and a protocol previously described by Wasiak *et al*. (2002) [61]. Extracted peptides were then subjected to LC-MS/MS. First, peptides were injected into a 300 μm × 5 mm Zorbax C18 trapping column (Agilent Technologies, Mississauga, Ontario, Canada) and peptides were subsequently resolved on a 10 cm × 75 micron PicoFrit column (New Objective, Inc., Woburn, Massachusetts, USA) containing C18 packing (BioBasic, Inc., Markham, Ontario, Canada). Peptides were eluted from the column with a 15 min gradient of 10-95% acetonitrile (v/v) containing 0.1% formic acid (v/v) at a flow rate of 200 nl/min using an Agilent 1100 series NanoHPLC system (Agilent Technologies). Eluted peptides were electrosprayed as they exited the column. Mass spectrometric data were acquired on a QTRAP 4000 (Applied Biosystems, Streetsville, ON, Canada) using the Information Dependent Analysis feature of Analyst 1.4.1 software (Applied Biosystems). Briefly, up to three double, triple or quadruple charged ions of intensity greater than 2 × 10^6 ^counts per sec from each enhanced-MS survey scan were selected for passage into a collision cell. Collision-induced dissociation was facilitated by collision with nitrogen gas; fragment ions were trapped in quadrupole (Q)3 and scanned. Three enhanced-product ion scans at a speed of 4000 atomic mass unit/sec from 70 to 1700 m/z were averaged for each selected precursor ion.

The tandem mass spectrometry raw data were transferred from the QTrap 4000 linear ion trap mass spectrometer to a server and automatically manipulated for generation of peak lists by employing the Distiller version 2.1.0.0 software http://www.matrixscience.com/distiller.html. The peak-listed data were then searched against the National Center for Biotechnology Information non-redundant database ftp://ftp.ncbi.nlm.nih.gov/blast/db/ downloaded October 6, 2008) using the Mascot search engine version 2.1.04 http://www.matrixscience.com. The search was limited to the Mammalia taxonomy (Taxonomy ID 40674; 707975 sequences; 279723342 residues).

### Statistical analyses

#### Cytokines and Haptoglobin

The cytokine responses for each subject was assessed using the estimated area under the curve described by the change in response from days 1-6 compared to baseline. All data points were adjusted for baseline values by and subtracting day 0 readouts. These values were then log-transformed prior to analysis in order to stabilize the variance. The general linear model procedure was used to perform a statistical analysis of these estimates in addition to the change in response from the baseline (day 0) for days 14 and 21 pi, using SAS (SAS Institute Inc., Cary, North Carolina, USA). The Tukey-Kramer adjustment was used to perform pair-wise comparisons of the 3 treatments. Data are presented as the least square mean of the log-transformed values ± standard error of the mean. For haptoglobin, repeated measurements taken over the course of 24 hours were analyzed using mixed models which included the 3 treatment groups as well the interaction between time and group, according to the method described by Wang and Goonewardene (2004) [62]. Changes were determined using day 0 as baseline values. The fixed effects were the 3 treatment groups, days and the interaction between treatment and day, and subject within treatment group was included as a random effect in the model. The MIXED procedure from SAS (SAS Institute Inc.) was used for analysis. Linear and quadratic orthogonal polynomial contrasts over time were used to assess differences in the responses between treatment groups. Data are presented as the least square mean of the log-transformed values ± standard error of the mean.

#### Milk proteomics

The experimental design was a completely randomized design with three treatments of *S. aureus *and repeated measurements for each subject at days 0, 2 and 14. All raw values (spot intensity) were log transformed in order to stabilize the variances, and baseline (day 0) values were subtracted from days 2 and 14. The general linear model (GLM) procedure was used to assess differential expression of each protein spot between treatment groups on days 2 and 14 pi, relative to the average of day 0 values using SAS (SAS Institute Inc., Cary, NC, USA). Multiple comparisons between the 3 treatment groups were performed using Tukey-Kramer's adjustment. P-values from each comparison were then corrected based on the false discovery rate (FDR) adjustment using the MULTTEST procedure. Data are presented as the least square mean of the log transformed values ± standard error of the mean.

## Authors' contributions

YK coordinated and performed experiments. HA performed the animal infections and cultured the bacteria. BM, CR, and NK designed and coordinated the project. All authors drafted the manuscript and approved its content.
